# Demographic features and prognostic factors of uterine carcinosarcoma: a study at a specialized center in Saudi Arabia

**DOI:** 10.3389/fonc.2025.1594233

**Published:** 2025-09-05

**Authors:** Wala Mehros, Sarah Aldosari, Rahaf Alharbi, Wasan Alshareef, Wesal Hawsawi, Halah Fallata, Hatim Aljifree, Alaa Althubaiti

**Affiliations:** ^1^ Department of Oncology King Abdul-Aziz Medical City, Ministry of The National Guard Health Affairs, Jeddah, Saudi Arabia; ^2^ King Abdullah International Medical Research Center, Jeddah, Saudi Arabia; ^3^ Department of Obstetrics and gynecology, College of Medicine, King Saud bin Abdulaziz, University for Health Sciences, Jeddah, Saudi Arabia; ^4^ Department of Basic Medical Sciences, College of Medicine, King Saud bin Abdulaziz, University for Health Sciences, Jeddah, Saudi Arabia

**Keywords:** uterine carcinosarcoma, overall survival, demographics, prognosis, endometrial neoplasm

## Abstract

**Objectives:**

Uterine carcinosarcoma is a rare, aggressive biphasic tumor comprising both carcinomatous and sarcomatous elements. The overall prognosis of women with uterine carcinosarcoma is poor, with a median overall survival of less than two years. The predictors of survival for patients with uterine carcinosarcoma in the Kingdom of Saudi Arabia have not yet been fully elucidated; this study therefore explored the demographic features and prognostic factors of uterine carcinosarcoma.

**Methods:**

This A cross-sectional study was conducted among all confirmed carcinosarcoma cases at Princess Noorah Oncology Center, National Guard Hospital, Jeddah, Saudi Arabia, from January 2003 to December 2023. Data were collected on demographic features, medical history, stage, treatment modality, and disease outcome.

**Results:**

A total of 34 patients with carcinosarcoma were identified, accounting for 4.7% of all endometrial cancer cases. Sixty percent of patients were diagnosed early, during stages 2B and below. The most common presentation was post-menopausal bleeding, occurring in 90% of the sample. Kaplan–Meir analysis revealed an overall median survival of 14 months.

**Conclusion:**

The findings confirmed the aggressiveness of the tumor. Late tumor stage was identified as a factor affecting patients’ survival and outcome, being associated with poor prognosis and short survival time.

## 1 Introduction

Uterine carcinosarcoma (UCS), also known as malignant mixed Müllerian tumor, is a biphasic tumor characterized by the presence of both carcinomatous (epithelial) and sarcomatous (stromal tissue) elements. The carcinomatous element can be either low- or high-grade endometrial cancer, whereas the sarcomatous element can be either homologous (containing cell types normally found in the uterus, such as stromal sarcoma and fibrosarcoma) or heterologous (consisting of other components, such as rhabdomyosarcoma, osteosarcoma, and liposarcoma) (1, 2). Although the underlying pathogenesis of UCS remains unclear, numerous molecular studies have shown that, rather than having two independent progenitors, both elements originate from a carcinoma lineage that undergoes sarcomatous dedifferentiation (3–5).

UCS is a rare tumor representing approximately 5% of all endometrial cancers (6). While it has been perceived as a disease of the elderly, rates of UCS in younger patients appear to be increasing in recent decades (7). Black women have an increased risk compared to other ethnicities (8, 9), and exposure to tamoxifen and pelvic radiation are some of the recognized risk factors for developing UCS (10–12). A stage shift has been noted in recent years, with increasing nodal metastasis and decreasing distant metastasis (13). Unfortunately, the overall prognosis of women with UCS is poor, with a median overall survival (OS) of less than two years and a five-year OS of 33.4% during the period from 1975 to 2012 (14). It has also been shown that cases exhibiting sarcoma dominance (where the sarcoma component makes up more than 50% of the tumor) are generally associated with a worse prognosis (15). Due to its rarity, specified treatment guidelines for UCS are lacking. The standard treatment approach for those with operable tumors is hysterectomy-based surgery, with a noted increase in the inclusion of chemotherapy (24% to 65.8% from 1988 to 2016) and a decrease in the utilization of external radiotherapy (30.6% to 21.6% from 2005 to 2016) (16–18).

Despite the increasing worldwide knowledge on the topic of UCS, it remains understudied, with an absence of recent publications focusing solely on the Kingdom of Saudi Arabia. Obtaining data from a Saudi Arabian population is crucial as their characteristics differ from those of other studied populations. This study therefore aimed to explore the demographic features and prognostic factors of uterine carcinosarcoma at a specialized center in Saudi Arabia.

## 2 Methods

### 2.1 Study design, participants, and settings

This cross-sectional study was conducted over a period of 20 years at Princess Noorah Oncology Center, National Guard Hospital, Jeddah, Saudi Arabia, among patients with uterine carcinosarcoma.

The study included all female patients diagnosed with uterine carcinosarcoma at the hospital from January 2003 to December 2023. Patients with other types of uterine cancer were excluded. Sample size calculations were not applied since all patients presenting with uterine carcinosarcoma over the past 20 years were included. A consecutive sampling technique was implemented, and 34 patients met the criteria.

### 2.2 Data collection

A chart review was conducted to extract patient data from electronic health records maintained in the BESTCare system, a comprehensive health information platform used at our institution. In alignment with institutional standards at King Abdullah International Medical Research Center (KAIMRC), all observational studies, including this one, adhere to the STROBE (Strengthening the Reporting of Observational Studies in Epidemiology) guidelines.

The extracted data were categorized into demographic information (including age, age at diagnosis, body mass index [BMI, kg/m²], and nationality) and relevant medical history (such as smoking status, hypertension, diabetes mellitus, use of hormone replacement therapy, family history of malignancy, and postmenopausal bleeding). Prognostic factors included patient status, type of surgical procedure, receipt of chemotherapy or radiation therapy, recurrence, and tumor stage, which was assessed according to the International Federation of Gynecology and Obstetrics (FIGO) staging system (18).

### 2.3 Ethical considerations

The study was approved by the Institutional Review Board at King Abdullah International Medical Research Center (IRB SP23J/009/02).

### 2.4 Data analysis

Statistical analyses were performed using JMP software (John’s Macintosh Project), version 10.0 (SAS Institute Inc., Cary, NC, USA). Categorical data, such as the treatment type, smoking status, and comorbidities, were presented as frequency and percentage. Non-parametric approaches were used to evaluate numerical data (age). Kaplan–Meier survival analysis was used to determine the median survival for each stage.

## 3 Results

Of the 723 patients diagnosed with endometrial cancers over the study period, only 34 were found to have carcinosarcoma. The median age of these patients was 67 IQR (38 - 96), and the median age at diagnosis was 63.5 years (IQR: 33 – 90). The BMI distribution in the dataset demonstrates that only two of the patients were classified as underweight, with a BMI below 18.5 kg/m^2^. Five patients fell within the healthy BMI range of 18.5 to 24.9, while 11 individuals were categorized as overweight, with a BMI between 25 and 29.9. Sixteen patients were classified as obese, with a BMI of 30 or higher, making up the majority of the sample.

For most of the patients (90%), post-menopausal bleeding was the first manifestation of carcinosarcoma. Eight patients had a family history of malignancy, while the other 23 did not report any such history. With respect to comorbidities, 23 of the patients had diabetes, and 26 had hypertension, constituting 74% and 79% of the sample, respectively. **Table 1** presents the demographics of the participants.

**Table 1:** Patients' demographics and medical history.

Variable	Descriptive
Age (years), median and range (min– max)	67 (38 _ 96)
Age at diagnosis (years), median and range (min– max)	63.5 (33 _90)
BMI, N (%)	
Below 18.5 (underweight)	2(6)
18.5_24.9 (healthy)	5(15)
25_29.9 (overweight)	11(32)
30 and above (obese)	16(47)
Smoking, N (%)	
Yes	1(3)
No	28(97)
Post-menopausal bleeding, N (%)	
Yes	27(90)
No	3(10)
Family history of malignancy, N (%)	
Yes	8(26)
No	23(74)
Diabetes, N (%)	
Yes	23(74)
No	8(26)
Hypertension, N (%)	
Yes	26(79)
No	7(21)
Hormonal Replacement therapy, N (%)	
Yes	4(20)
No	16(80)

Continuous data presented as median and range (min_max). Categorical data presented as number (percentage). BMI, body mass index.

**Figure 1:**
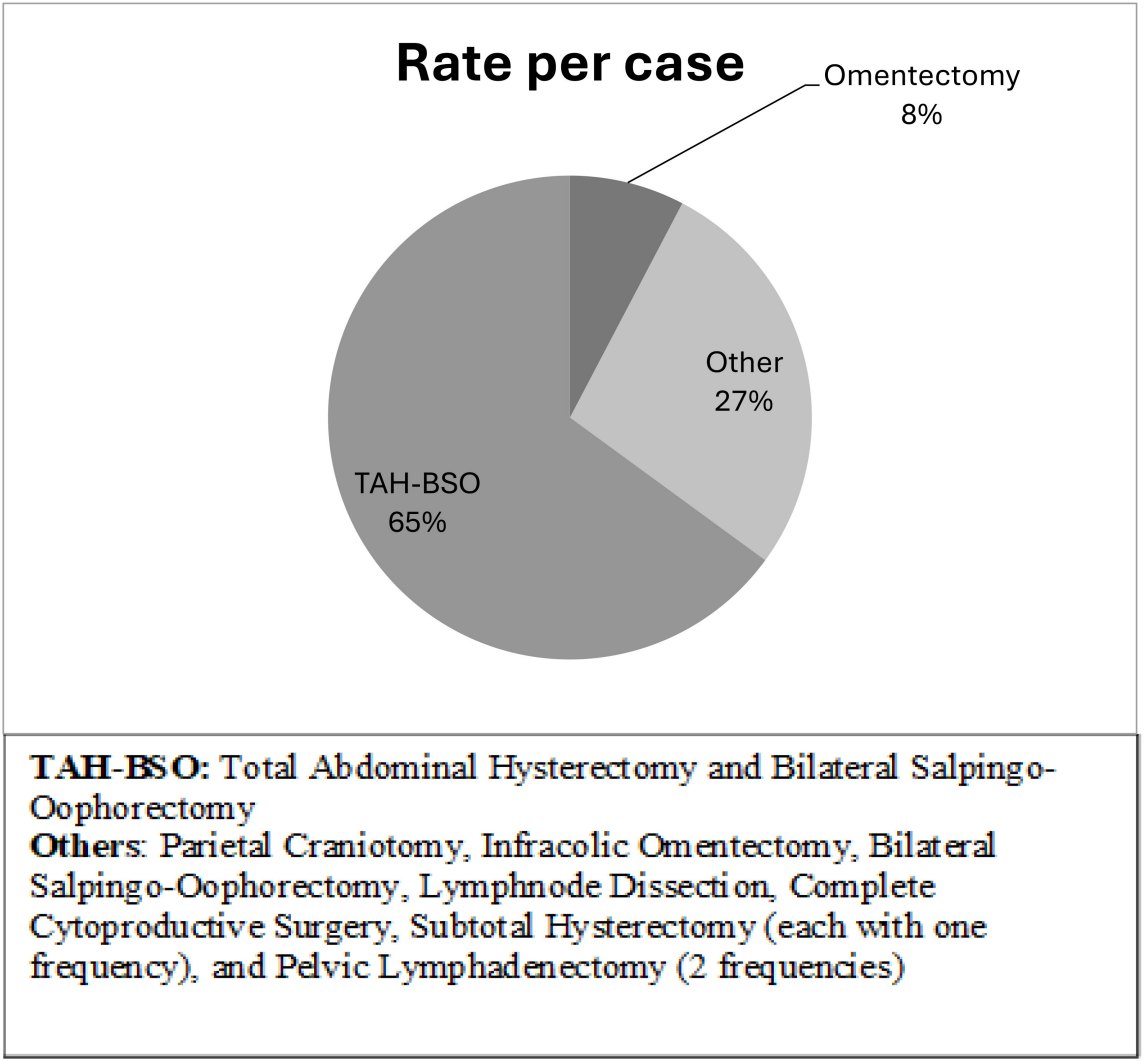
Surgical Procedure.

**Figure 2:**
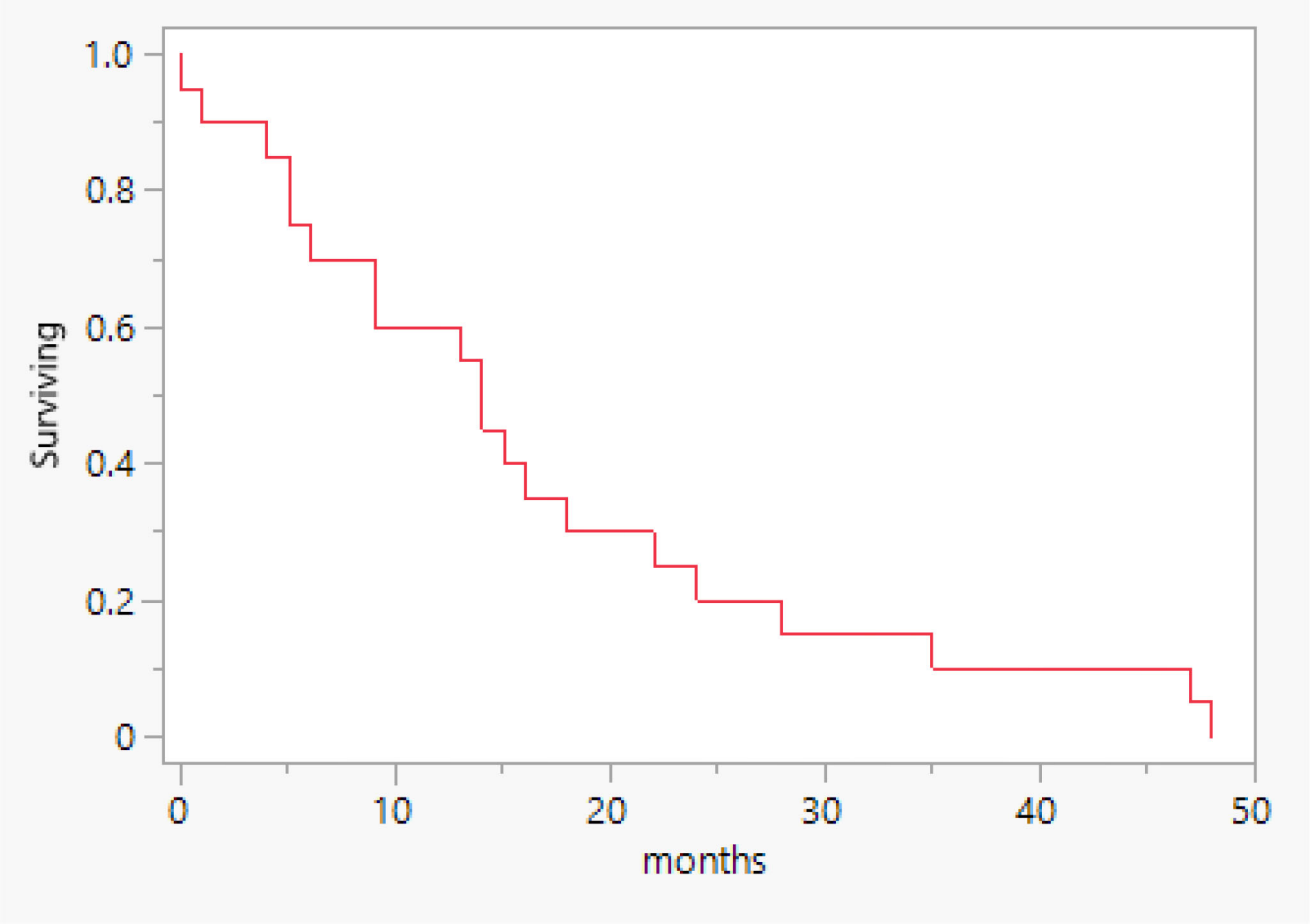
Overall survival.

**Figure 3:**
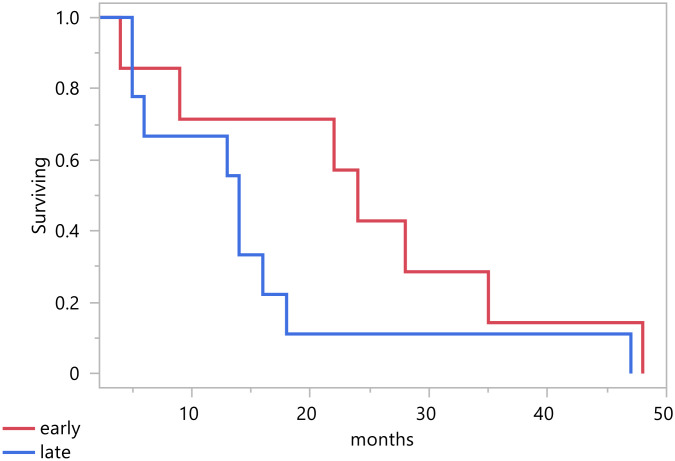
Survival per stage.

As shown in **Table 2**, the tumor stage distribution indicates that 18 patients (60%) were diagnosed with an early-stage tumor, defined as FIGO stage 2B and below. In contrast, 16 patients (40%) presented with a late-stage tumor, classified as FIGO stage 3A and above. This distribution highlights a substantial number of cases detected at an advanced stage. In terms of treatment modalities, 82% of the patients underwent a surgical procedure, the most common of which was total abdominal hysterectomy with bilateral salpingo-oophorectomy, accounting for 65% of surgeries (**Figure 1**). In addition, 59% (20) received chemotherapy, and 68% (23) underwent radiation therapy. A pivotal factor is the patient’s survival status, revealing that 21 patients passed away, and the total median survival was 14 months (range 5 – 22 months) (**Figure 2**). **Figure 3** illustrates that the median survival was 24 months (range 4 – 35 months) for the early stage (FIGO stage 2B and below) and 14 months (range 5 – 18 months) for the late stage (FIGO stage 3A and above).

**Table 2:** Tumor stage, treatment modality, recurrence, and current patient status.

Variable	Descriptive
Tumor stage(early/late), N (%)	
Early (FIGO stage 2B and below)	18(60)
Late (FIGO stage 3A and above)	16(40)
Surgical procedure, N (%)	
Yes	28 (82)
No	6 (18)
Chemotherapy in total, N (%)	
Yes	20 (59)
No	14 (41)
Chemotherapy alone, N (%)	
Yes	6(18)
No	28(82)
Radiation therapy in total, N (%)	
Yes	23(68)
No	11(32)
Radiotherapy alone, N (%)	
Yes	9(26)
No	25(74)
Both chemotherapy and radiotherapy, N (%)	
Yes	14(41)
No	20(59)
Recurrence, N (%)	
Yes	14(41)
No	17(55)
Current patient status, N (%)	
Decreased	21(62)
In-remission	9(26)
On treatment	2(6)
Loss of follow up	2(6)

Categorical data presented as number (percentage). FIGO, International Fedration of Gynecology and Obstetrics.

## 4 Discussion

This study found that UCS accounted for 4.7% of the endometrial cancer cases at Princess Noorah Oncology Center between January 2003 and December 2023. This finding is consistent with the results of a study in the United States between 1973 and 2013 in which UCS was diagnosed in 11,000 (4.7%) of 235,849 endometrial cancer cases, as well as the worldwide prevalence (13, 19). The synchronous prevalence implies a unified epidemiological pattern across distinct geographical areas, different healthcare systems, or demographic configuration.

According to Bansal et al., females with this type of cancer tend to be older, with a median age of 70 years. This is closely aligned with our sample’s median age of 67 (20). However, UCS’ age of onset is exhibiting a shift toward a younger demographic. Our study found that the median age at diagnosis was 63.5, consistent with the findings of similar studies (13, 14). This shift may indicate the progression of risk factors of the disease or improvements in its early detection and diagnosis. In addition, previous studies on uterine carcinosarcoma have demonstrated that older age and advanced stage at diagnosis are important prognostic factors (21).

The majority of the patients in our study were overweight and obese, accounting for 32% and 47%, respectively. Obesity has been found to increase the risk of aggressive subtypes of endometrial tumors such as carcinosarcoma (22). The increasing prevalence of obesity in younger age groups may be a possible explanation for the rising incidence of UCS among young women (20). These findings emphasize the importance of understanding the complex pathological mechanisms contributing to obesity, which will provide insights into its association with UCS that can guide the development of effective management and prevention strategies.

Our results demonstrate a median survival of 14 months among our sample. This is lower than that reported by previous studies (21 months) (23) but is comparable to the 16-months median survival found by Kotowicz et al. (24). This notable variation in survival periods might be explained by the small sample size. The mortality rate of our sample was 62%, comparable to the rate of 89% recorded by Kotowicz et al. (24). Although UCS accounts for < 5% of all uterine malignancies, it is responsible for > 15% of uterine cancer-associated deaths (19). Compared with grade 3 endometrial tumors, the mortality risk associated with UCS was found to be 45% less (20). These significantly high death rates and short survival times are indicative of the aggressive nature of the disease, as well as the increased disease burden. A possible explanation for the high mortality rate in our study is the older age of our patients. In their study, Bansal et al. Noted a 61% increase in mortality among women with carcinosarcoma aged 60 years or above compared with patients aged 40 – 60 years (20). This finding focuses our attention on illustrating age-stratified management and suggesting proper prevention measures. Even though the overall survival is poor, the stage of the disease at diagnosis is a predictive factor (21, 25).

In our study, the median survival rates for early and late stages were 24 months and 14 months, respectively. This indicates that diagnosis in the early stage of the disease is a predictor of improved survival (6, 20). Early detection allows for earlier intervention, underscoring the importance of screening and adopting effective preventative interventions. Another predictor of better survival outcomes is receiving radiation therapy (20). In particular, intervention with radiation therapy in combination with chemotherapy can reduce the risk of recurrence (14). In our group of patients, 6 (18%) received chemotherapy alone, 9 (26%) received radiation alone, and 14 (41%) received both chemotherapy and radiotherapy. Furthermore, 55% of the patients did not experience a recurrence, illustrating the potential benefits of appropriate treatment.

Recent advances in molecular oncology have significantly enhanced the understanding of endometrial cancer biology. The Cancer Genome Atlas (TCGA) proposed a molecular classification system dividing endometrial cancers into four subtypes: POLE-ultramutated, microsatellite instability-high (MSI-H), copy-number low (CN-low), and copy-number high (CN-high) (26). This system has proven to be a reliable predictor of prognosis and treatment response, as demonstrated in a pooled analysis of over 2,800 patients (27). Notably, uterine carcinosarcoma (UCS) is most frequently associated with the CN-high subtype (53.2%), which correlates with poor prognosis, while MSI-H and POLE-ultramutated subtypes are less common (26).

These molecular distinctions have therapeutic implications. Immune checkpoint inhibitors (ICIs) have demonstrated clinical benefit, especially in endometrial cancers displaying MSI-H or POLE-ultramutated profiles, underscoring the importance of identifying these subtypes in clinical practice (28, 29). Additionally, HER2/neu overexpression has been reported in a subset of UCS cases, and targeted therapies have shown promising activity, even in tumors with low HER2 expression (30, 31).

Unfortunately, molecular profiling—including assessment of HER2, MMR status, and POLE/TP53 mutations—was not routinely available at our center during the study period. This limited our ability to explore the molecular landscape and therapeutic targets in our cohort. Future studies incorporating comprehensive molecular characterization are essential to improving risk stratification and guiding personalized treatment strategies in UCS.

This study has several limitations. Its single-center design may limit the generalizability of the findings to broader populations, and the retrospective nature of the study introduces potential for selection bias due to missing or incomplete clinical data. Additionally, the small sample size renders the study underpowered to detect small differences; therefore, p-values and confidence intervals were not reported as definitive indicators of statistical significance. The absence of a comparison group further limits the contextualization of results, and the lack of molecular profiling restricts the ability to explore key prognostic biomarkers. Despite these limitations, this study represents the first comprehensive characterization of uterine carcinosarcoma in Saudi Arabia and provides a valuable foundation for future multicenter, hypothesis-driven research.

## 5 Conclusions

This study investigated the demographic features and prognostic factors of uterine carcinosarcoma—a rare tumor that tends to affect older females—in Saudi Arabia. We identified advanced tumor stage as a factor affecting patients’ survival and outcome due to its association with poor prognosis and short survival time. Conversely, diagnosis of this malignancy in the early stages leads to a better prognosis. Further research in the field is needed to enhance our findings and evaluate other features of the patient demographics and disease outcomes.

## Data Availability

The raw data supporting the conclusions of this article will be made available by the authors, without undue reservation.
